# Bio–Cultural Diversities: Why They Matter Now

**DOI:** 10.3390/biology11030475

**Published:** 2022-03-21

**Authors:** Andrea Pieroni

**Affiliations:** 1 University of Gastronomic Sciences, Piazza Vittorio Emanuele II 9, 12042 Pollenzo, Italy; 2 Department of Medical Analysis, Tishk International University, Erbil 44001, Iraq

The time at which we write these lines is a dramatic time of war. The Russian invasion of Ukraine seriously re-addresses the importance of studying and assessing bio–cultural diversities, but also of celebrating and even advocating for their dynamic preservation; this richness may, in fact, represent one of the most potent means of fostering respect for others’ cultures, social sustainability, and therefore, lasting peace.

Bio–cultural diversities, or eco-diversities [1], are the focus of this Special Issue. We started from the reflection that, in the current Anthropocene dominated by an unprecedented global and climate-change-centered crisis, biodiversity is threatened to possibly a greater extent than ever before in human history; we reflected that promoting and fostering its truly sustainable use require tight collaborations with those human communities who still retain complex conglomerates of traditional/local environmental knowledge, practices, and beliefs (LEK). The current SI focuses on the links that exist between biodiversity and cultural diversity (including linguistic diversity) and shows how these two dimensions are interconnected and interdependent. In fact, global goals to reduce the rate of biodiversity loss have not been achieved for the most part. Even some examples of conservation success show that losses can be halted and even reversed [2]. On the other hand, biodiversity loss parallels the tremendous loss of linguistic and cultural diversities, with homogenization, boosted by economic globalization, being a key element in modernity [3]. Moreover, biodiversity hotspots sometimes seem to be associateed with areas where linguistic diversity continues to flourish [4]. This SI presents inspiring ethnobotanical and economic–botanical contributions focusing on the mutual interdependence of plant resources and cultures, as well as the potentially important economic impacts of these resources on the implementation of sustainable development trajectories [5].

Using linguistic [6], cross-cultural ethnographic [7,8,9,10], and historical [11] approaches, the contributions to this SI show that bio–cultural diversities are complex, co-evolving assemblages that are threatened now more than ever. Standardizing centripetal strengths operated by several cultural and economic processes and actors may be responsible; one of these processes may be generated by the centralized organization of illiberal states, as one contribution to the SI very elegantly demonstrates [12].

Indeed, eco-cultural diversities need to be recognized and evaluated by the scientific community, and their evolution, complexities, and fascinating aesthetics require further research in the years to come. Celebration of the immense diversity of life in all of its forms is particularly essential across educational platforms, in order to train new generations of citizens and scholars and avoid repetition of the tragedies that are currently unfolding before our eyes.
